# Neurology

**Published:** 1905-03-25

**Authors:** 


### PROGRESS IN MEDICINE AND SURGERY.

### NEUROLOGY.

**Tactile Sensation.**—In an address on the paths of sensory conduction in the peripheral and central nervous system Horsley[1] said that if a peripheral nerve be divided the resulting area of anæsthesia is divided by a sharp line from the surrounding sensitive skin, there being no zone along the border of the anæsthetic area in which there is a slight loss of sensation. There is thus no evidence of overlapping in the distribution of peripheral nerves; the anæsthesia is absolute and bounded by a sharp line. In transverse lesions of the spinal cord, *e g.* from fracture, there is absolute anæsthesia of the skin supplied by the cord below the lesion, but at the upper edge of this there is a zone of a certain breadth in which there is either paræsthesia or diminished response to tactile sensations. If the lesion be acute there is actually hyperæsthesia above the zone of absolute anæsthesia. There is only one explanation of these facts, that the grey matter is concerned in carrying upwards afferent impressions and in the neighbourhood of the lesion is impaired to a certain extent. When the lesion is in its acute stage the grey matter is not only partially damaged but is also highly congested, hence the hyperæsthesia at the upper border of the anæsthesia. In the base of the brain sensory impressions pass upwards in the fillet. For some time it was doubtful whether these fibres all terminate in the thalamus or whether some, at any rate, pass upwards, without being interrupted by grey matter, to the cortex. The experiments of Mott and of Horsley have shown that the former view is the true one; and correspondingly it is found, clinically, that lesions of the fillet produce an anæsthesia of the cord type—*i.e.*, absolute in the part that is cut off from the higher brain. From the thalamus go fibres to all parts of the cortex, including the Rolandic area. Hence it is that, coupled with every destruction of the motor region of the cortex producing localised paralysis, we have also localised paralysis of sensation and touch. The patient has loss, to a slight degree, of actual sensation of touch, and he loses knowledge of the exact position of that touch. It is the loss of this knowledge of the position of the tactile impression which produces incoördination. In examining a patient it is not sufficient to touch him and say, "Did you feel that?" but the part to be tested should be screened from the patient, it should be touched lightly, and he should be compelled to mark with his own index finger the part touched. If the patient, though perhaps only partially anæsthetic, is unable to localise the touch, the lesion must be above the thalamus.

**Hemiplegia in Cerebral Tumour.**—Three cases have been published by Williamson[2] showing that hemiplegia of gradual onset, in which weeks or a few months elapse before the paralysis is complete, may be regarded as strong evidence of cerebral tumour, even when optic neuritis is absent, or when optic neuritis, headache, and vomiting are all absent. Optic neuritis is present in about four-fifths of all cases of brain-tumour; in about one-fifth of the cases it is absent, and in these cases the diagnosis is more difficult. The hemiplegia of cerebral tumour is not invariably of gradual onset; it may in rare cases be rapid, especially if there should be hæmorrhage around or within the growth. But when it does occur of gradual onset there is no single sign of greater value in the diagnosis.

**Insanity and Epilepsy.**—The influence of these diseases on the expectation of life, and so their relation to life assurance is discussed by Gowers,[3] who expressed the opinion that a family history of either insanity or epilepsy or both, as a rule, should not depress a life below the average if the proposer is over 20 years of age. Exceptions existed—for instance, a family tendency might be so strong and manifested so definitely in collaterals of the same generation as to compel its recognition. In the case of the actual proposer, a previous attack of insanity should as a rule preclude acceptance. Previous attacks of epilepsy are generally equivalent to the present existence of the disease. Some cases may be accepted, of course, at a considerable extra, if they have nearly reached middle life, and the character of the disease has shown no tendency to become more grave. General paralysis of the insane apparently causes ten times as many premature deaths as all the other forms of insanity put together. Any proposer who presents any suggestive symptoms, however uncertain, should be rejected. In determining this, the presence or absence of the knee-jerks and light pupil reflex should be tested, and the past history in relation to syphilis ascertained.

**Hysteria and Neurasthenia.**—Ascherson[4] reports several cases of hysteria and neurasthenia, and gives an interesting summary of the characteristics of the two states and of the theories of their causation. Neurasthenia is a condition characterised by a varied symptom-complex, physical and mental, irreconcilable with our preconceived notion of any other clinical disease. The subjective aspect of the condition is the predominant, and is in no sort of harmony with what we are able to determine objectively. The condition mingles itself with the individuality of the patient and stamps it with timidity, pessimism, and mental depression. The objective characters all take the form of some kind of incompleteness of function, especially when a practical issue is at stake. Hysteria differs from neurasthenia in that its objective symptoms are at least equal to, if not in excess of, its subjective symptom, and may be in some cases the only ones apparent; these symptoms often simulate other diseases, though differing in their inconstancy. The condition is rather paroxysmal than chronic, and has *per se* very little effect on the character of the patients. Janet's explanation of neurasthenia is that the highest psychological level to which the human being is capable is only reached by those who possess the power of perceiving impressions clearly and of elevating them into the sphere of their consciousness to such an extent as to make them a part of reality. These two faculties are denied to the neurasthenic, who, recognising the inability, becomes the subject of pessimism and fatigue. But because his mental activities must find vent somehow, they do so in ways which are satisfied by lower forms of energy, expressed clinically in attacks of anguish and emotionalism, tics, and the various obsessions to which he is liable. Janet advances two hypotheses to explain hysteria—there exist two separate centres, conscious and subconscious, in which the operations of the mind take place, the functions of which are identical to the extent that they can both receive, store, recall, and act upon impressions from without, but differ in this respect, that whereas the functions of the higher centre are appreciated consciously by the individual, those of the other take place without his being aware of them. In health, while we make use of either of these centres in our mental operations, we always use the higher centre in dealing with new impressions. In the hysterical individual there is a tendency for impressions from without to be dealt with only by the lower centre, a tendency to lapse into automatism; and the breaches produced thereby in the sum of his past experiences, give rise to what is known as the disaggregation of his personality.

**Congenital Word-blindness.**—Ten additional cases of this condition have been recorded by Stephenson,[5] who defines it as a congenital one not necessarily associated with any other defect (mental or physical) where the learning of words or letters from printed or written characters is difficult or, it may be, even impossible. It is probably due to a congenital defect in the usual memory centre for words and letters. The ages varied from 7 to 23, and the cases were mostly in males. Vision was generally normal; refraction was emmetropic once and hypermetropic with or without astigmatism in the remaining nine cases. General intelligence was good. The auditory memory was highly developed in most of the patients. Considerable improvement can be expected with special training, and may take place comparatively quickly, especially when begun early. The diagnosis offers few difficulties if the fact be grasped that there is such a thing as congenital inability to read.

**Arterio-Sclerosis of the Spinal Cord.**—Collins and Zabriskie[6] record two cases of this condition which they say is usually diagnosticated according to its terminal condition — principally chronic myelitis, occasionally spinal apoplexy, and, more rarely still, acute myelitis. The first patient, aged 51, gradually developed, during the course of some months, the following symptoms: weakness and easily induced fatigue of the legs; peculiar sensations in the lower extremities described as "jerky," "numbness," and "heavy"; progressive incontinence of urine; progressive paraplegia. The objective symptoms were atypical, the tendon jerks were absent, and a Babinski phenomenon was present, and with these there were slight disorders of sensibility, paraplegia, and trophic manifestations. The patient died from cardiac weakness. It was found that the arteries and veins everywhere throughout the cord and brain were in process of arterio-sclerosis, especially marked in the spinal cord. There were hæmorrhages in the right posterior horn and left anterior horn in the ninth dorsal segment. In the anterior horns in the same segment the cells were in a state of chromatolysis. From this situation ascending and descending degenerations in the white matter could be traced. This condition can be diagnosed during life, especially if there are symptoms, subjective or objective, pointing to generalised arterio-sclerosis. For instance, a man aged 49, complained of weakness in the legs, especially after a little exertion, and a tendency to incontinence of urine. There was a moderate degree of sclerosis of the peripheral blood-vessels with heightened blood-pressure, and a perceptible amount of weakness of the legs, though with normal tendon-jerks and flexor plantar reflexes. The patient underwent great improvement while taking small doses of nitroglycerin and iodide, and being treated with rest, warm baths and massage.

[1] Pract., Nov. 1904 [2] Ibid. [3] Lancet, Oct. 15, 1904. [4] Pract., Sept. 1904. [5] Lancet, Sept. 19, 1904. [6] Med. Rec., Sept. 3, 1904.

